# Prevalence of HPV in Mexican Patients with Head and Neck Squamous Carcinoma and Identification of Potential Prognostic Biomarkers

**DOI:** 10.3390/cancers13225602

**Published:** 2021-11-09

**Authors:** Galo Méndez-Matías, Cindy Velázquez-Velázquez, Rosario Castro-Oropeza, Alejandra Mantilla-Morales, Diana Ocampo-Sandoval, Ana Burgos-González, Carlos Heredia-Gutiérrez, Isabel Alvarado-Cabrero, Rosa Sánchez-Sandoval, Abigail Barco-Bazán, Fátima Chilaca-Rosas, Patricia Piña-Sánchez

**Affiliations:** 1 Molecular Oncology Laboratory, Oncology Research Unit, Oncology Hospital, National Medical Center, Mexican Institute of Social Security, Mexico City 06720, Mexico; galo_scholermann@hotmail.com (G.M.-M.); cindyk.velazquez@gmail.com (C.V.-V.); rosariocastrooropeza@gmail.com (R.C.-O.); diannaocsand@gmail.com (D.O.-S.); roosesanzbio@gmail.com (R.S.-S.); abigail.barco.92@gmail.com (A.B.-B.); 2 Biological Sciences Program, Graduate Studies, UNAM, Mexico City 04510, Mexico; 3 Pathology Department, Oncology Hospital, National Medical Center, Mexican Institute of Social Security, Mexico City 06720, Mexico; alemantimora@yahoo.com.mx (A.M.-M.); keme2.tijax12@gmail.com (I.A.-C.); 4 Radiation Oncology Department, Oncology Hospital, National Medical Center, Mexican Institute of Social Security, Mexico City 06720, Mexico; ana_14157@hotmail.com (A.B.-G.); jcheredia97@hotmail.com (C.H.-G.); fatychro@hotmail.com (F.C.-R.)

**Keywords:** head and neck squamous cancer, human papillomavirus, Mexico, differential expression, biomarkers, prognosis

## Abstract

**Simple Summary:**

Head and neck squamous cell carcinomas (HNSCC) are a heterogeneous group of neoplasms that show diverse clinical and biological characteristics associated with human papillomavirus (HPV). Biological and clinical characterization is essential to stratify patients based on prognostic and predictive factors. The biological features of HNSCC may change according to geography and population characteristics. Studies on the molecular biology of HNSCC in Mexico are scarce. In the present study, we analyzed 414 Mexican patients with HNSCC and determined the presence and genotype of HPV, p16 expression, and global gene expression profiles. Twenty-two percent of total cases were HPV+, and 32% were p16+. We identified genes associated with survival, such as SLIRP, KLF10, AREG, ACT1, and LIMA. In addition, CSF1R, MYC, and SRC genes were identified as potential therapeutic targets. This study offers information that may be relevant for our understanding of the biology of HNSCC and the development of therapeutic strategies.

**Abstract:**

Head and neck squamous cell carcinomas (HNSCC) show a variety of biological and clinical characteristics that could depend on the association with the human papillomavirus (HPV). Biological and clinical characterization is essential to stratify patients based on prognostic and predictive factors. Reports on HNSCC are scarce in Mexico. Herein, we analyzed 414 Mexican patients with HNSCC, including oropharynx (OPSCC), larynx (LASCC), and oral cavity (OCSCC), and identified HPV DNA and p16 expression. Global gene expression profiles were analyzed in 25 HPV+/p16+ vs. HPV−/p16− cases. We found 32.3% p16+ and 22.3% HPV+ samples, HPV 16, 18, 39, 52, and 31 being the most frequent genotypes. For OPSCC, LASCC and OCSCC, 39.2, 14.7, and 9.6% were HPV+/p16+, respectively. High expression of SLIRP, KLF10, AREG, and LIMA was associated with poor survival; in contrast, high expression of MYB and SYCP2 correlated with better survival. In HPV+ cases, high expression of SLC25A39 and GJB2 was associated with poor survival. Likewise, EGFR, IL-1, IL-6, JAK-STAT, WNT, NOTCH, and ESR1 signaling pathways were downregulated in HPV+ cases. CSF1R, MYC, and SRC genes were identified as key hubs and therapeutic targets. Our study offers information regarding the molecular and clinical characteristics of HNSCC in Mexican patients.

## 1. Introduction

Head and neck squamous cell carcinomas (HNSCC) are the seventh most common cancers worldwide, with approximately 890,000 new cases and mortality of 450,000 reported in 2018 [1]. HNSCC are heterogeneous, not only in terms of their anatomical site but also in terms of their pathologic, molecular, clinical, etiologic, and geographic traits [2,3,4,5]. It is currently recognized that some of these carcinomas are related to infection by the human papillomavirus (HPV) and the transformation it induces [6]. Interestingly, increasing evidence has shown that HNSCC associated with HPV has a better prognosis, especially in oropharynx squamous cancers (OPSCC) [7,8]. An increase in HNSCC associated with HPV and decrease in cases related to smoking have been documented both in Europe and North America [7]. The presence of HPV in HNSCC varies with the anatomical site; that is to say, it has been found in 48.5% of OPSCC, 24% of cancers of the oral cavity (OCSCC), and 22% of those in the larynx (LASCC) [9]. The most frequent viral genotype in HNSCC is HPV16, followed by HPV 18, 33, 35, 52, 45, and 39 [10].

Evaluation of p16 expression or detection of viral transcripts is currently recommended, in addition to viral DNA detection, to identify HPV−related cases in HNSCC [11]. In fact, the eighth edition of The American Joint Committee on Cancer recommends identifying HPV−positive cases through p16 as a surrogate marker in OPSCC staging [12]. Likewise, several studies have analyzed global expression profiles for the molecular classification of HNSCC [13,14,15,16], although validation and clinical application are still in progress.

It is noteworthy that most reports on the biological and clinical features of HNSCC come from countries with a very high development index, and only a few reports exist that present data from middle/low-income countries. Indeed, in Mexico, there are only a few studies regarding the relation between HPV and HNSCC, and they are limited to assessing the presence of HPV in a small number of samples [17,18,19,20]. Thus, in trying to contribute to our knowledge on the biology of HNSCC and its relation to clinical traits, especially in developing countries, such as Mexico, in the present study, we have generated information on the molecular characteristics of HNCSS in 414 Mexican patients. We have focused on identifying cases associated with HPV based on detection of viral DNA and p16 overexpression, and we have determined transcriptional profiles to identify differentially expressed genes linked to prognosis.

## 2. Materials and Methods

### 2.1. Sample Selection

All cases included in this study were collected at the Unidad Médica de Alta Especialidad de Oncología del Centro Médico Nacional Siglo XXI, of the Instituto Mexicano del Seguro Social (Mexican Institute of Social Security), the largest center for cancer treatment in Mexico and a national reference center. Cases diagnosed as HNSCC during the period 2011–2017 were identified from records from the Pathology Department. In total, 414 cases of OPSCC, LASCC, and OCSCC with available clinical information and biological samples were selected. Clinical characteristics such as sex, age, stage, and location were recorded. The project was approved by the Local Committee for Research and Ethics in Research (number R-2013-3602-14, and R-2017-3602-31).

### 2.2. P16 Expression

Histologic sections (4 μm thick) were taken and mounted on electrocharged slides (VWR). Immunohistochemistry with antibody CINtec^®^ p16 Histology (clone E6H4™) was performed to detect p16. A Master Polymer Plus Detection System (Peroxidase) was used as per the instructions of the manufacturer. Slides were dyed with hematoxylin, dehydrated, and mounted on synthetic resin. Tissues were observed with a DM750 Leica microscope and classified as positive when at least 70% of tumor cells had intense nuclear or cytoplasmic immunoreaction [21].

### 2.3. HPV Detection and Genotypification

Histological sections stained with hematoxylin–eosin were used to select those containing representative areas for further study. Two to four 10 μm sections were taken for DNA extraction with Promega ReliaPrep™ FFPE gDNA Miniprep System kit. In addition, Epoch™ Microplate Spectrophotometer (BioTek Instruments, Inc.; Winooski, VT, USA) was used to quantify 2 µL of DNA.

In all p16 positive cases, HPV detection and genotypification were performed by Inno Lipa INNO-LiPA^®^ HPV Genotyping Extra II (Fujirebio; Zwijnaarde, Belgium) as per the manufacturer’s instructions. This method identifies 32 viral genotypes by PCR of a 62 bp fragment from the HPV L1 region and reverse hybridization. Results were interpreted with Liras^®^ soft LIPA HPV V2 software. Likewise, HPV Direct Flow CHIP kit (Master Diagnóstica, Granada, Spain), which identifies 35 viral genotypes from a 150 bp region in gene L1 that is amplified by PCR, was also used. Reverse hybridization was performed automatically with hybriSpot 24TM equipment. Additionally, cases negative for p16 were assessed by PCR and electrophoresis with primer set from Inno Lipa or HPV Chip Direct Flow Chip, while respective reverse hybridization was performed on positive cases. Only those genotypes that could be identified by both tests were considered for genotype analysis (30 genotypes).

### 2.4. HTA 2.0 Expression Microarrays

To delve into the molecular differences between HPV+/p16+ and HPV−/p16− HNSCC patients, we performed a genome-wide expression analysis in selected Formalin-fixed paraffin-embedded (FFPE) samples using the HTA 2.0 Affymetrix platform. The samples selected for RNA extraction with RNeasy FFPE Kit (QIAGEN), according to the manufacturer’s instructions, had at least 70% tumor cells. DNA was quantified with PicoGreen. A Sensation Plus kit was used with an RNA concentration between 20 and 50 ng/µL. Eleven OCSCC cases, twelve LASCC cases, and three OPSCC cases were analyzed, of which 7 cases were HPV+/p16+, and 18 cases were HPV−/p16−. (Supplementary Table S1).

Gene expression was evaluated using the Human Transcriptome Array 2.0 microarray (HTA 2.0, Affymetrix) which allows the analysis of 44,699 protein-coding transcripts and 22,829 non-coding transcripts. Data were deposited in the Gene Expression Omnibus (GEO submission GSE 181805). Data were normalized by the RMA (Robust Multichip Average) method and analyzed using the Affymetrix Transcriptome and Expression Analysis Console (TAC 4.0, Applied Biosystems). All samples analyzed approved the quality parameters set by the TAC console. A fold change greater than 1.5 or less than −1.5 and a *p*-value ≤ 0.05 were used to compare gene expression between HPV+/p16+ and HNSCC HPV−/p16− HNSCC samples. To verify gene identity and annotation, we used the Integrative Genomics Viewer (IGV) and the Bioconductor biomaRt package. Differentially expressed genes were employed for further analysis.

### 2.5. Survival Analysis

Correlation between HNSCC patient survival and expression of differentially expressed genes was analyzed using software that integrates gene expression and clinical data (KMplot). Samples were grouped according to the expression of each gene (low vs. high expression) using auto-select best cutoff. A total of 500 HNSCC samples from The Pan-cancer RNA-seq section was used for mRNA analysis. For miRNA analysis, the miRpower section of 523 HNSCC patients was used to generate each Kaplan–Meier survival graph. The hazard ratio with 95% confidence and long rank *p*-value was calculated for each gene.

### 2.6. In Silico Validation

RNAseq, clinicopathological, and survival data of TCGA-HNSCC patients were downloaded and visualized from the UCSC Xena browser platform [22]. Gene expression data were analyzed to verify the association between DE genes among HPV+ and HPV− HNSCC patients. Information on patients with HPV status was corroborated in the cBioPortal; patients without HPV status information were discarded.

### 2.7. Enrichment Analysis

To identify the main signaling pathways, biological processes, and “master” regulators altered in HPV−positive HNSCC patients, the differentially expressed genes were used and filtered in MetaCore (version 20.4.70300) and Key Pathway Advisor (KPA) version 17.4 software suites. All results met the threshold of enrichment value of *p* < 0.05 and FDR < 0.05.

### 2.8. Heatmap

The expression levels of DEG in Mexican HNSCC patients were pictured in a heatmap using ClustVis, a modified version of the heatmap R package (version 0.7.7). Samples were clustered in an unsupervised heatmap using Euclidean correlation distances.

### 2.9. Statistical Analysis

Clinical data, p16 expression, HPV detection, and genotypification were processed with IBM SPSS Statistics Version 24. Qualitative variables were described as frequencies and percentages. The relation with clinical parameters was assessed by chi-square test and Fisher’s exact test. GraphPad Prism 8.0 software was used for plotting graphs, and the statistical analysis employed was Welch’s *t*-test of unequal variances.

## 3. Results

### 3.1. Clinical Data

The analysis included 414 cases for which there was a representative biological sample for HPV and p16 assessment. Age was registered in 365 cases, with a mean of 65.0 ± 12.8 years and a range of 27–95 years. In 24.1% of patients, diagnosis occurred at 55 years of age or less; 71.3% of cases were males. Regarding anatomical location, 19.6% of cases occurred in the oropharynx, 41.2% in the oral cavity, and 39.1% were identified as primary tumors of the larynx. Likewise, 65% of cases were diagnosed in advanced stages (III and IV), and the predominant histological grade was moderately differentiated in 66.6% of cases. Smoking was reported in 64.4% of cases, and 61.5% of patients consumed alcohol (Table 1).

### 3.2. Presence of HPV and P16

Among the 414 cases registered, p16 was evaluated in 412 cases and HPV in 368 because of insufficient tissue in some samples or lack of internal control amplification. Furthermore, 32.3% (*n* = 133) were positive for p16, while 22.3% (*n* = 82) were positive for one of the HPV genotypes. Prevalence of HPV and p16 was significantly different with respect to age as prevalence was greater in younger patients (Table 1). Where both markers were assessed, concordance was 82.7%, and the lowest concordance was found in patients older than 76 years (*k* = 0.43). HPV was detected in 40.5% of OPSCC, 17.2% of OCSCC and 18.2% of LASCC (Table 1).

**Figure 1:**
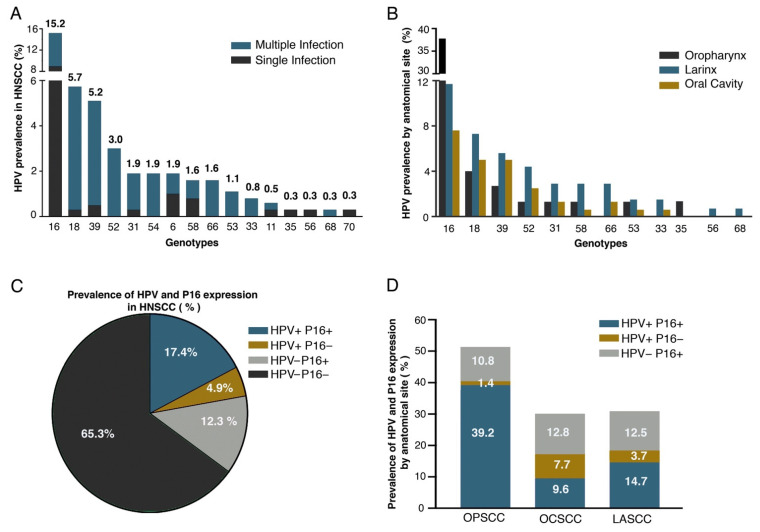
Prevalence of HPV and p16 in HNSCC. (**A**) Genotype prevalence in HNSCC. Cases found with each genotype are shown as single infection (SI) and multiple infections (MI); (**B**) distribution of most frequent high-risk genotypes per anatomical site; (**C**) prevalence of HNSCC according to HPV presence and p16 expression; (**D**) prevalence of HPV and p16 expression per anatomical site. Concordance in OPSCC was *k* = 0.75, in OCSS *k* = 0.36 and in LSCC *k* = 0.54, significance 0.000.

Sixteen different genotypes were identified, 61% of which were single infections, and 38.5% of the cases showed multiple genotypes (Figure 1A). HPV35, 56, and 70 were infrequent and present as single infections, while genotypes 33, 52, 54, 66, and 68 were only found together with other genotypes (Figure 1A). In all three anatomical locations, HPV16 and HPV18 were the most frequent, and in OPSCC, HPV16 was found in 37.8% of cases. HPV 18, 39, and 52 were more often found in non-OPSCC, whereas HPV 31, 58, and 66 were more frequent in LASCC (Figure 1B). Among all HNSCC cases analyzed, 17.5% were HPV+/p16+ (Figure 1C); 39.2, 9.6, and 14.7% of OPSCC, OCSCC and LASCC cases, respectively, were HPV+/p16+. The highest prevalence of HPV+/p16− was found in OCSCC (7.7%) (Figure 1D). Only age and anatomical location were associated with HPV+/p16+ (Table 2).

### 3.3. Expression Analysis

**Figure 2:**
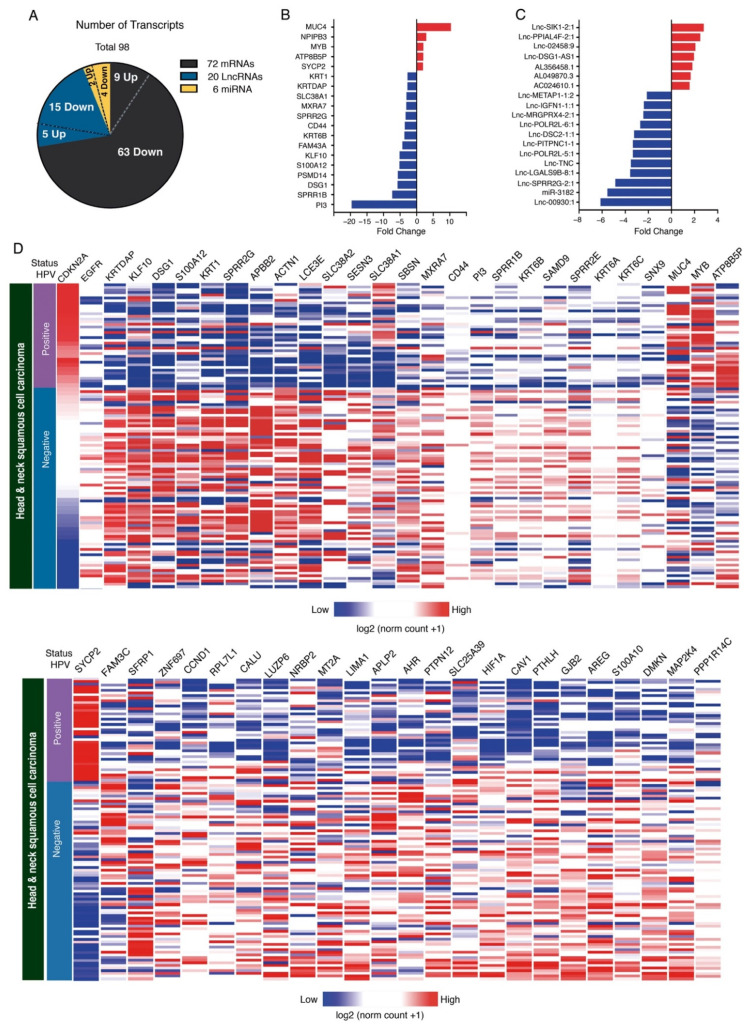
Transcriptome changes in HNSCC patients associated with presence or absence of HPV and p16. (**A**) Graph shows the classification of differentially expressed transcripts in HPV+/p16+ samples versus HPV−/p16− samples in tissues with HNSCC. Main differentially expressed mRNAs (**B**) and lncRNAs (**C**) in HPV+/p16+ vs. HPV−/p16− samples. (**D**) Heat map showing gene expression between HPV+ tissues (*n* = 39) and HPV− tissues (*n* = 75) in HNSCC patients. Columns show the expression of each transcript validated in TCGA samples. Gene expression is shown in a range from red (greater) to blue (lesser).

Expression analysis showed changes in the expression of various coding and non-coding RNAs. However, since knowledge in the transcriptomic context is continually improved and updated, we used an IGV (Integrative Genomics Viewer) to perform a careful analysis of the location and genetic classification of differentially expressed genes, thus verifying gene identity. Using this tool, we were able to identify 98 annotated transcripts from the 56 initially available with a fold change greater than 1.5 or less than −1.5 (Supplementary Figure S1). We performed an unsupervised heatmap analysis exhibiting the expression levels of significant coding and non-coding transcripts in Mexican HPV+/p16+ and HPV−/p16− patients. All fold changes of coding transcripts in our Mexican cohort are shown in Supplementary Figure S2. As shown in Figure 2A, differential expression analysis identified 16 upregulated genes and 82 downregulated genes between the HPV+/p16+ versus the HPV−/p16− samples (Figure 2A).

**Figure 3:**
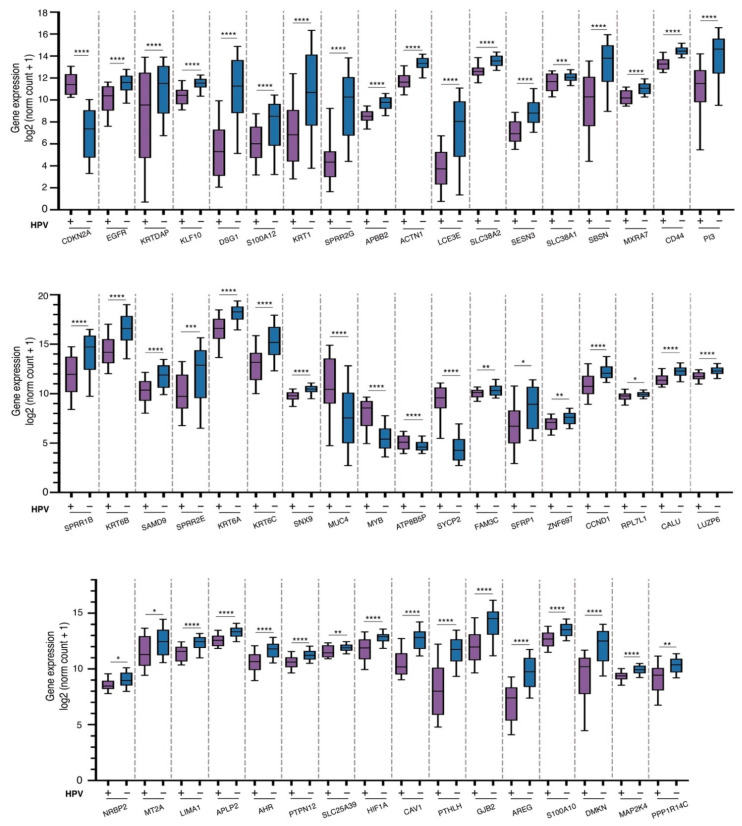
Expression profiles of differentially expressed mRNA validated in HNSCC samples from TCGA. Graph shows measure of expression profile of DE mRNA between HPV+ (purple) and HPV− (blue) tissues. Statistical analysis by Welch’s *t*-test (**** *p* < 0.0001, *** *p* < 0.001, ** *p*<0.01 and * *p* < 0.05).

Of these, 73.4% were mRNAs, and 26.5% were non-coding RNAs. Figure 2B shows the top DE mRNA genes across HPV+/p16+ vs. HPV−/p16− HNSCC patients. Regarding non-coding transcripts, we found 20 differentially expressed lncRNAs and six miRNA; the top ones are shown in Figure 2C. To further support our microarray data, we analyzed changes in the expression of DE mRNAs on the UCSC Xena platform [22]. We used a database of 604 preloaded TCGA samples from patients with head and neck cancer. Only samples with known HPV status were considered. We obtained 114 samples; 39 of them were HPV+, and 75 were HPV−. The expression of DE mRNAs between HPV+/p+16 vs. HPV−/p16− HNSCC samples was compared, and results showed that 51 out of 72 DE genes were successfully validated. Supplementary Table S2 shows the main characteristics of the mRNAs differentially expressed, fold change, validation in the TCGA database, and their roles in cancer. In addition, 47 mRNAs were significantly downregulated in HPV+ HNSCC patients, including KLF10, DSG1, SPRR2G, ACTN1, CCND1, HIF1A, and AREG (Figure 2D and Figure 3). We also found four significantly upregulated mRNAs (MUC4, MYB, ATP8B5P, and SYCP2) in HPV+ vs. HPV− HNSCC tissues (Figure 2D and Figure 3). These findings collectively support the expression changes found in our samples.

**Figure 4:**
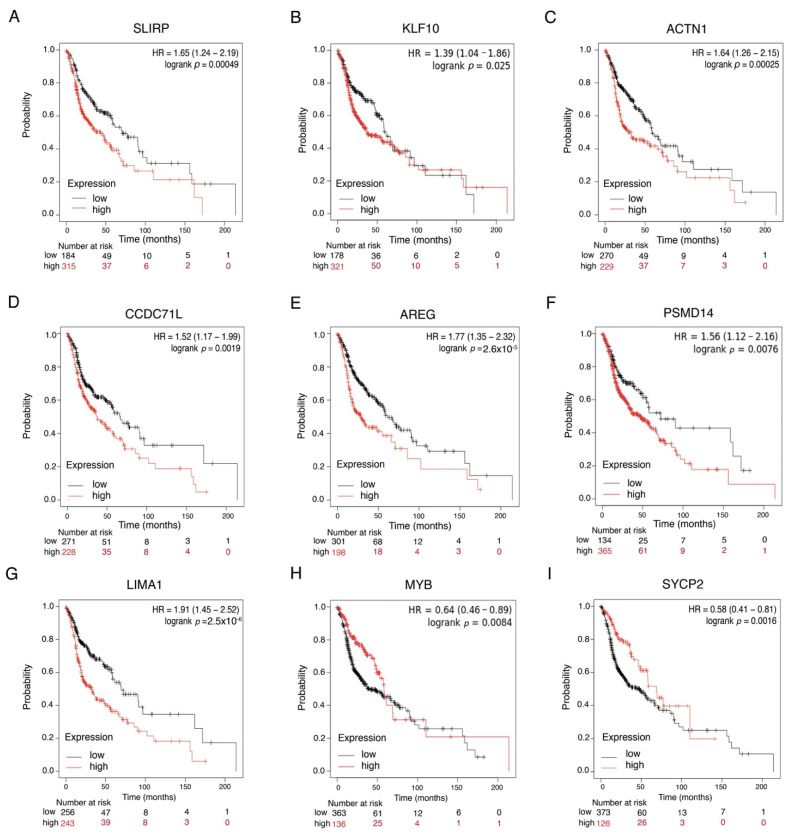
Differentially expressed mRNAs show clinical importance in patients with HNSCC. (**A**–**I**) Kaplan–Meier curves comparing global survival with respect to expression of DE SRLIP, KLF10, ACTN1, CCDC71L, AREG, PSMD14, LIMA1, MYB, and SYCP2 genes from patients with HNSCC. *p* values are shown in each graph. Survival data were analyzed with respect to database from Pan-cancer RNAseq Data.

We then sought to determine the clinical relevance of differentially expressed genes in HNSCC. We searched Pan-cancer RNAseq Data at KmPlot and compared the association between gene expression and overall survival using data from 500 HNSCC patients. The results indicate that 43 of the 72 mRNAs analyzed were related to survival. Patients with low expression of SLIPRP, KLF10, ACTN1, CCDC71L, AREG, PSMD14, and LIMA had better survival than those with high expression (Figure 4A–G), while high expression of MYB and SYCP2 was associated with better survival (Figure 4H–I). Other DE genes, such as CD44, EGFR, CAV1, and CCND1, were also associated to survival (data not shown). In addition, using the HNSCC miRpower database of 523 patients, we found that patients with low expression of miR-3182 or miR-103a2 also had better survival (data not shown). Taken together, these data suggest that expression of DEG is linked to better prognosis and could be associated with HPV+ HNSCC patients.

**Figure 5:**
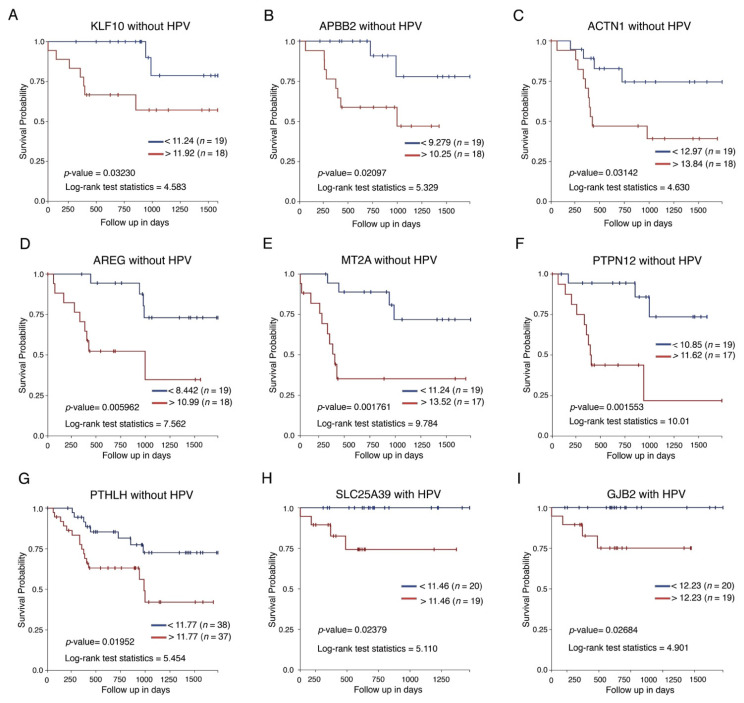
Differentially expressed mRNAs show clinical importance in patients with HNSCC according to HPV status. (**A**–**G**) Kaplan–Meier curves comparing global survival with respect to expression of DE genes in HPV negative samples (KLF10, APBB2, ACTN1, AREG, MT2A, PRPN12, and PTHLH. (**H**,**I**) Kaplan–Meier curves comparing global survival in relation to DE genes in HPV+ samples, but not samples without viral infection (SLC25A39 and GJB2). *p* values are shown in each graph.

Given that expression of these genes may be involved with different clinical outcomes, we analyzed HPV+ or HPV− patient survival data and mRNA expression. The results indicated that high expression of KLF10, APBB2, ACTN1, AREG, MT2A, PTPN12, and PTHLH was associated with low survival in HPV−unrelated HNSCC (Figure 5A–G), whereas high expression of SLC25A39 and GJB2 was associated with poor survival only in HPV+ HNSCC (Figure 5H,I).

### 3.4. Analysis of Signaling Pathways and Molecular Processes

**Figure 6:**
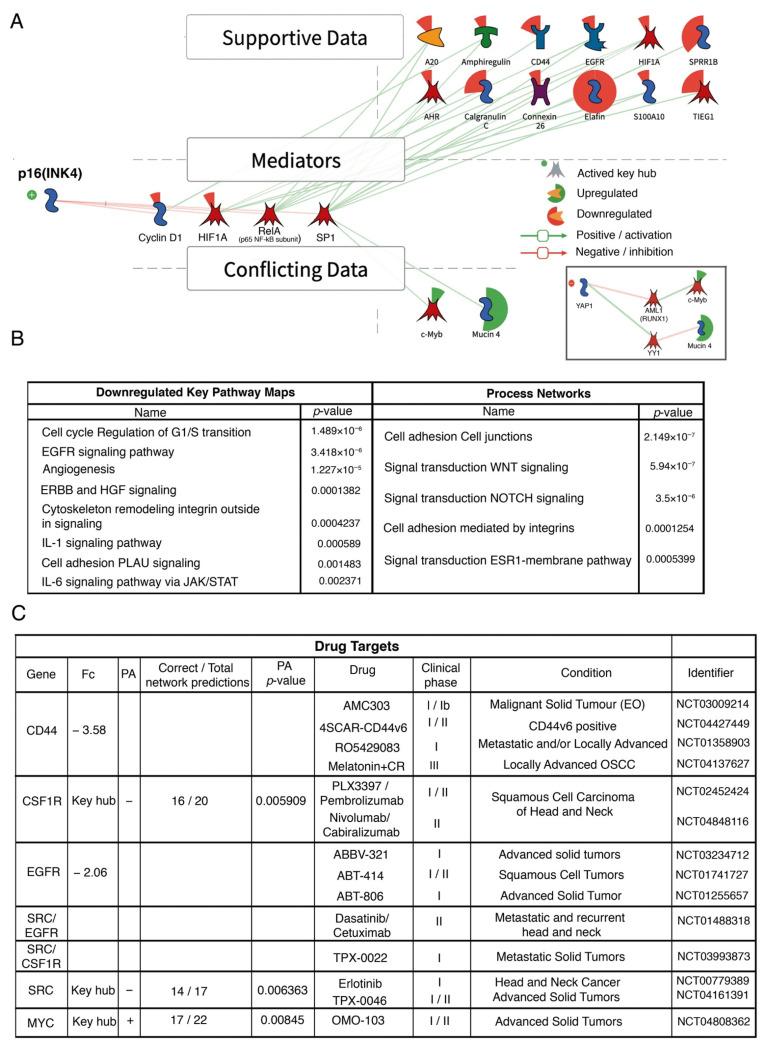
Integration map, signaling pathways, and molecular processes enriched in HPV+ HNSCC patients. (**A**) Diagrammatic summary suggesting p16 activation as a “master” regulator in HPV−positive patients. As shown, p16 negatively regulates key mediators such as cyclin D1 or HIF1A to modify the transcription of gene characteristics of the HPV+ phenotype. Supportive data refers to expression data consistent with the signaling pathway regulated by the key hub p16. The conflicting data are differentially expressed transcripts whose expression is not explained by p16; thus, they may be regulated by different pathways such as the YAP/TAZ signaling route. (**B**) Main significantly enriched signaling pathways with inhibited status, as well as altered molecular processes in HPV+ samples (joint *p* values ≤ 0.001). Data produced from transcripts with differential expression greater and lesser than 1.5. Molecules in a red background proportionally indicate inhibition in HPV− samples. (**C**) Identification of potential drug targets and ongoing clinical trials.

To find signaling pathways and biological functions involved in changes in expression in HPV+/p16+ patients, differentially expressed genes were assessed. KPA analysis revealed p16 activation (INK4) and its relevance as an outstanding key hub in HPV−positive patients (Figure 6). Negative regulation of intermediary products such as cyclin D1, HIF1A, RELA, and SP1 was observed, while genes such as CD44, EGFR, HIF1A, PI3 (elafin), and SPRR1B, among others, showed negative regulation caused by mediator inhibition.

MetaCore and KPA allowed the identification of 23 pathways that were significantly downregulated. Figure 6B presents the eight most significant pathways: ERBB and HGF, cell cycle, EGFR signaling, angiogenesis, cytoskeleton remodeling integrins, IL-1 signaling pathway, cell adhesion, and IL-6 signaling via JAK/STAT. Regarding molecular pathways with the most changes in expression, we observed NOTCH, WNT, and estrogen receptor 1 (ESR1) transduction pathways, as well as cell adhesion processes (Figure 6B). Additionally, KPA analysis revealed CD44 and EGFR as therapeutic targets, as well as CSF1R, MYC, and SRC as key hubs, all of which are involved in various clinical trials (Figure 6C).

## 4. Discussion

Several European and North American groups have confirmed that a significant number of HNSCC cases are associated with HPV and that these have a better prognosis than HPV− cases [23,24]. In addition, mutation, expression [13,14,15,16], and to a lesser degree, epigenetic profiles have been characterized in HNSCC. Studies on biological features of HNSCC in Mexico are few and limited to the report of the presence of viral DNA in a limited number of cases. Studies on HNSCC in Latin America are also uncommon [25,26] The present study is the largest regarding the number of cases reported in Mexico and the second largest in Latin America [27]. This is also the first Mexican study to include a transcriptomic analysis.

In keeping with different reports [10,26], HPV16 was the most frequent genotype found in this study, followed by HPV18. It should be noted that our study identified greater diversity and frequency of other genotypes, such as HPV 39 and 52, in LASCC (Figure 1B), than the ones observed in previous reports [27]. Interestingly, in a Brazilian study, only 4.1% of OPSCC cases were associated with HPV16, in clear contrast with the data reported in our study and throughout the world [2]. Apart from HPV16, the majority of the most frequent genotypes occurred jointly with other genotypes. This has also been observed in cervical cancer [28]. Nowadays, the clinical and biological relevance of multiple infections in HNSCC is not completely understood; thus, this is an area for future research.

We found that from all HPV+ cases, 78% (64/82) were also positive for p16. This association was particularly evident in OPSCC (96.7% of HPV+ cases were also p16+). In agreement with previous reports, 39.2% of OPSCC were HPV+/16+ [9,29]. Other studies reported 22.4 [30] and 30% based on the presence of viral RNA [26]. Albers and collaborators reported 45% of OPSCC cases as HPV+/p16+, and this resulted as the group with the highest survival rate [31]. In our study, OPSCC was the anatomical site with the lowest HPV+/p16− prevalence (1.3%), while OCSS had the highest (7.7%). In this regard, 7% of HPV+ OPSCC cases had a deletion in CDKN2A, and in non-OPSCC, the presence of deletions was greater (14.2%) [32]. We also found that OCSCC was the anatomical site with the lowest concordance between HPV and p16 (*k* 0.36). Previous studies showed HPV in 31% and p16 in 30% of OCSCC cases; nevertheless, no correlation was found (*k* = 0.1) nor relation with survival [33].

A study in northern Mexico found a 47% prevalence of HPV in LASCC [34]. This is much higher than the levels reported globally, i.e., 22 and 5.7% [9,30]. In our study, 18.4% of LASCC were HPV+ and only 14.7% were HPV+/p16+. These molecular differences could be due to population differences within the national territory or methodological issues in the definition of HPV positivity. In non-OPCSS, 4% of cases have been attributed to HPV [32]. Our study identified 12% HPV+/p16+ in non-OPSCC, although the greatest discrepancy between HPV presence and p16 expression was recognized in OCSCC.

It should be noted that 13% of HNSCC cases were HPV−/p16+. In addition, p16 has been identified as a senescence marker. Thus, it is interesting to observe that nearly 70% of the HPV−/p16+ patients in the present study were over 60 years of age. The importance of p16 expression in HNSCC has been revealed in various studies. Vidal Loustau et al. identified an association between p16 expression and survival in OCSS, even in the absence of HPV [35]. Likewise, Padhi and collaborators reported that a low p16 expression or CDKN2A deletion was associated to recurrence, poor prognosis, and low survival rate in OCSCC [36]. Lechner et al. found p16 expression similar in both HPV− and HPV+ cases, particularly in non-OPSCC. This overexpression is mainly due to mutations in NSD1 [37], although mutations in RB1 and CDKN2A have also been associated to p16 overexpression [38]. The group led by Bryant has reported that p16 expression has the same prognostic impact in non-OPSCC as in OPSCC [39]. In contrast, Tabliabued et al. found that HPV is related to better prognosis only in OPSCC [29], while Larque reported p16 overexpression due to mutations in CDKN2A in HPV− LASCC cases associated to worse prognosis [40]. Because of such diverse findings, it is necessary to take a closer look at the relation between HPV and the anatomical site and its clinical impact in relation to other risk factors, such as alcoholism and smoking, as well as to identify clinically relevant biomarkers in HNSCC, especially concerning the presence or absence of HPV.

Several studies have focused on HNSCC expression profiles identifying molecular groups with an impact on prognosis [13,14,15,16]. Other studies have compared expression profiles between HPV−positive and HPV−negative cases [41,42,43] Among all the patients included in the present study, we determined the expression profile in selected cases based on presence of HPV and p16 expression. Seventy percent of DE genes were validated in the UCSC Xena platform database. Among the DE genes, we identified PTHLH, CAV1, CCND1, SYCP2, and MYB, which have been previously reported in HNSCC [43]. Specifically, SYCP2 overexpression has been relevant in HPV+ HNSCC [41,42,44]. Recent reports indicate that SYCP2 directly interacts with HP1α, avoiding its binding with H3K9me3, favoring dsDNA repair, and non-homologous end-joining by ATM [45]. On the other hand, HPV has been described to infect epithelial cells by CAV1-mediated endocytosis [46], and that HPV then induces a fall in CAV1 via p53/E6 inactivation [47]. Because of the constant overexpression of SYCP2, MYB and underexpression of CAV1 in HNSCC, these may be considered potential biomarkers of neoplasms associated to HPV, in addition to p16 and CCND1.

Other DE genes belonging to the proline-rich protein cluster (SPRR1B, 2E, and 2G); have also been reported with low expression [42]. In the present work, differential expression of genes such as APBB2, KLF10, SNX9, SLIRP, SLC38A1, and ZNF697 has been described for the first time. Additionally, our study identified 20 new IncRNAs in HNSCC (HPV+/p16+). In this regard, Nohata and collaborators identified 140 differentially expressed lncRNA in HPV+ tumors concerning HPV− [48]. Studies in the future must analyze the functional and clinical implications of lncRNA in HNSCC.

Advanced stage and HPV− HNSCCs have the worst prognosis and are the most frequent head and neck cancers, hence the importance of identifying biomarkers in these patients. In the present study, we identified that the genes with the most significant change rates, such as MUC4 and PI3, are not necessarily associated with prognosis; however, EGFR and CD44 were associated with global survival in HNSCC, confirming previous findings [49]. High CD44 expression is linked with poor prognosis, local recurrence, and metastasis in lymph nodes [50], although its prognostic significance has only been proven in OPSCC and LASCC, while there seems to be no correlation in OSCC [51]. Our analysis showed that high expression of AREG, an EGFR ligand, correlates with worse OS. In fact, AREG had greater significance in survival than EGFR (*p* = 0.000026 vs. *p* = 0.14). Overexpression of AREG has been related to lymphatic metastasis and is known to be negatively regulated by miR-34, which, in turn, is positively regulated by TP53 [52]. Although the HPV E6 oncoprotein inactivates TP53, it can have low functional expression levels and can even increase its stability by radiation [53]. Therefore, it is possible that the TP53-miR34-AREG axis is associated with decreased invasion and metastasis, and thus, with a better prognosis. Nevertheless, it should be noted that in patients with recurrent and metastatic HNSCC treated with cetuximab and chemotherapy, the greater benefit was observed in those with high AREG expression [54]. Therapeutic plans must therefore be adapted to the molecular profile of the patient to achieve the greatest possible benefit.

Our analysis shows that KLF10 overexpression in HNSCC correlates with an unfavorable prognosis, as in lung cancer [55]. KLF10 is a gene involved in a variety of signaling pathways and has been described as a potential prognostic marker in patients with oral cancer in early stages [56]. KLF10 binds to GC-rich DNA sequences to modulate activation or inhibition of transcripts involved in cell proliferation, inflammation, and apoptosis, among others [57]. On the other hand, ACTN1 expression was significantly associated with global survival, especially in HPV− cancers. It is worth noting that previous studies identified ACTN1 as part of genetic signatures in HPV+ HNSCC [58]. Thus, its expression could be a prognostic marker of survival in HPV− patients.

In our study, p16 was the most outstanding key hub. p16 is involved in regulation of the G1-S phase of the cell cycle; it inhibits cyclin-dependent kinase 4 (CDK4) and prevents Rb phosphorylation, leading to cell cycle deceleration [59,60,61]. Its overexpression has been widely documented in HPV+ neoplasms; therefore, our data support the relevance of p16 in GDE regulation in HNSCC. Apparently, positive regulation of c-Myb and MUC4 in HNSCC occurs by YAP inhibition more than regulation via SP1 (Figure 6A). Interestingly, the YAP/TAZ signaling pathway was significantly downregulated in our study (*p*-value = 0.0047). Likewise, YAP1 inhibition correlates with better prognosis and survival in OSCC [62]. It should be noted that negative regulation of these pathways supports a better prognosis in HNSCC patients.

We found that EGFR and CD44 were increased in HPV− cases. It is worth noting that both molecules are therapeutic targets. In this regard, it is relevant that cetuximab was the first targeted therapy approved for the treatment of HNSCC [63] and is currently the only clinically approved targeted therapy for this malignancy, although its use is limited [64]. Su et al. suggested that the benefit of EGFR inhibitors is probably restricted to p16+ cases and depends on the type of treatment [65]. Various clinical studies are currently being carried out in HNSCC with several EGFR inhibitors, especially in locally advanced disease, some in the context of HPV, as well as recurrence and metastasis [64].

Our analysis found three key hubs with differential expression (CSF1R, SRC, and MYC), which are responsible for networks of expression changes. CSF1R and SRC are upregulated in p16−/HPV− HNSCC and are candidates for therapeutic targets. In addition to the clinical trials in progress, drug combinations such as Dasatinib and CmpbA (IKKβ/NF-κB inhibitor) are being explored in HNSCC patients resistant to cisplatin [66]. On the other hand, MYC is overactivated in p16+/HPV+ HNSCC patients. MYC is considered a master gene and is one of the most well-studied genes in hematologic neoplasms and solid tumors. Furthermore, molecules have been recently developed that inhibit MYC directly or indirectly, such as OmoMYC and APTO-253, and are being assessed in preclinical and clinical trials. In the future, these could possibly be applied to neoplasms such as HPV+ HNSCC [67].

## 5. Conclusions

Our study brings further information regarding HPV prevalence in Mexican HNSCC patients based on HPV detection and p16 as a surrogate marker. The data presented herein represent the first gene expression profile in Mexican HNSCC patients comparing HPV+/p16+ versus HPV−/p16− and contribute to the potential identification of prognostic biomarkers and therapeutic targets in HNSCC. They also add to the finding of molecular pathways involved in HNSCC pathogenesis. Likewise, they show the viability of analyzing paraffin-embedded samples, which are a vast source of clinical and biological information. Undoubtedly, the identification of therapeutic targets, through approaches such as those presented in this study, will help determine the use of specific drugs in selected patients based on their biological traits and with the perspective of greater precision in the use of therapy.

## Figures and Tables

**Table 1:** Clinic characteristics of head and neck cancer patients, DNA HPV presence, and p16 expression.

Characteristics	Total		HPV+		P16+	
	%	*n* = 414	%	*n* = 368	%	*n* = 412
Age						
≤55	24.1	88	35.4	29	38.6	34
56–75	53.7	196	20.1	35	33.8	66
≥76	22.2	81	12.5	9	21.3	17
			*p* = 0.002		*p* = 0.043	
Gender						
Men	71.3	286	22.5	57	32.7	93
Woman	28.7	115	22.5	23	31.3	36
Anatomic site						
Larynx	39.1	162	18.2	25	30.4	49
Oropharynx	19.6	81	40.5	30	54.3	44
Oral cavity	41.3	171	17.2	27	23.5	40
			*p* = 0.000		*p* = 0.000	
Stage						
I–II	36.5	91	15.6	14	18.7	17
III–IV	63.5	158	20.1	30	32.3	51
					*p* = 0.26	
Histological Grade						
I	22.2	75	18.5	12	34.7	26
II	66.6	225	17.3	35	25.0	56
III	11.2	38	24.2	8	36.8	14
Smoke						
Yes	64.4	125	18.9	23	30.4	38
No	35.6	69	23.8	15	37.7	26
Alcohol						
Yes	61.5	112	19.1	21	30.4	34
No	38.5	70	14.3	9	31.4	22

HPV was evaluated in 368 cases and p16 in 412. The concordance between HPV and p16 was Kappa 0.55 *p* = 0.000.

**Table 2:** Distribution of cases HPV and p16 concordant and discordant and clinic variables associated.

Characteristics	HPV+/P16+		HPV−/P16−		Others		*p*
	%	*n*	%	*n*	%	*n*	
Age							0.019
≤55	26.8	22	53.7	44	19.5	16	
56–75	8.5	32	68.8	119	12.7	22	
≥76	8.5	6	76.1	54	15.5	11	
Gender							0.210
Men	18.7	47	67.3	169	13.9	35	
Woman	16.7	17	61.8	63	21.6	22	
Anatomic site							0.000
Larynx	14.7	20	69.1	94	16.2	22	
Oropharynx	39.2	29	48.6	36	12.2	9	
Oral cavity	9.6	15	69.9	109	20.5	32	
Stage							0.238
I–II	11.1	10	76.7	69	12.2	11	
III–IV	19.5	29	69.8	104	10.7	16	
Histological Grade							0.485
I	16.9	11	64.6	42	18.5	12	
II	11.9	24	72.6	146	15.4	31	
III	21.2	7	60.6	20	18.2	6	
Smoke							0.514
Yes	18.9.	23	71.3	87	9.8	12	
No	22.2	14	63.5	40	14.3	9	
Alcohol							0.803
Yes	18.2	20	70.0	77	11.8	13	
No	14.2	9	73.0	46	12.7	8	

## Data Availability

GEO submission GSE181805.

## Supplementary Materials

The following are available online at https://www.mdpi.com/article/10.3390/cancers13225602/s1, Figure S1: Recognition and classification of differentially expressed genes; Figure S2: Hierarchical clustering of differentially expressed genes in head and neck squamous cell carcinoma samples from Mexican patients; Table S1: Clinical characteristics of the samples analyzed for expression profiling; Table S2: Differentially Expressed Genes Characteristics.
