# Supplementary material for: Prevalence of HPV in Mexican Patients with Head and Neck Squamous Carcinoma and Identification of Potential Prognostic Biomarkers
Source: Cancers (Basel). 2021 Nov 9;13(22):5602. doi: 10.3390/cancers13225602 (PMC8616077; doi:10.3390/cancers13225602)
Supplement: Supplementary file 1 [file cancers-13-05602-s001.zip › Supplementary Table 1.pdf]

Supplementary Table S1. Clinical characteristics of the samples analyzed for expression profiling

CODE	GENDER	AGE	Anatomical Site	p16	HPV	STAGE	ALCOHOL	TOBACCO
HN070	F	55	Oral Cavity	Neg	Neg	IVA	No	No
HN087	M	49	Oral Cavity	Pos	16	IVA	No	No
HN182	M	NI	Larynx	Neg	Neg	IVA	No	No
HN183	M	66	Oral Cavity	Neg	Neg	III	No	No
HN184	M	63	Pharynx	Pos	16	IVA	Yes	No
HN185	M	NI	Larynx	Neg	Neg	IVA	Yes	Yes
HN186	F	36	Larynx	Pos	16	I	No	Yes
HN187	M	49	Pharynx	Pos	16	II	Yes	Yes
HN188	F	80	Oral Cavity	Neg	Neg	II	No	No
HN189	M	50	Larynx	Neg	Neg	IVA	Yes	Yes
HN193	M	68	Larynx	Neg	Neg	III	Yes	Yes
HN194	M	59	Larynx	Neg	Neg	III	No	No
HN195	M	NI	Oral Cavity	Neg	Neg	IVA	Yes	Yes
HN199	M	78	Larynx	Neg	Neg	III	Yes	Yes
HN200	M	80	Larynx	Neg	Neg	III	Yes	Yes
HN201	F	64	Pharynx	Pos	16	IVA	Yes	Yes
HN202	M	84	Pharynx	Neg	Neg	III	Yes	Yes
HN203	F	67	Oral Cavity	Pos	16	II	No	Yes
HN206	F	36	Oral Cavity	Neg	Neg	II	No	No
HN207	F	83	Larynx	Neg	Neg	III	Yes	No
HN209	M	52	Larynx	Pos	39	III	Yes	Yes
HN210	M	61	Larynx	Neg	Neg	NI	NI	NI
HN211	F	73	Oral Cavity	Neg	Neg	IVB	No	No
HN213	F	NI	Oral Cavity	Neg	Neg	NI	NI	NI
HN215	M	48	Oral Cavity	Neg	Neg	IVA	Yes	Yes

F: Female. M: Masculine. Neg: Negative. Pos: Positive. NI: Not identified
