# Supplementary figures and images for: Prevalence of HPV in Mexican Patients with Head and Neck Squamous Carcinoma and Identification of Potential Prognostic Biomarkers
Source: Cancers (Basel). 2021 Nov 9;13(22):5602. doi: 10.3390/cancers13225602 (PMC8616077; doi:10.3390/cancers13225602)

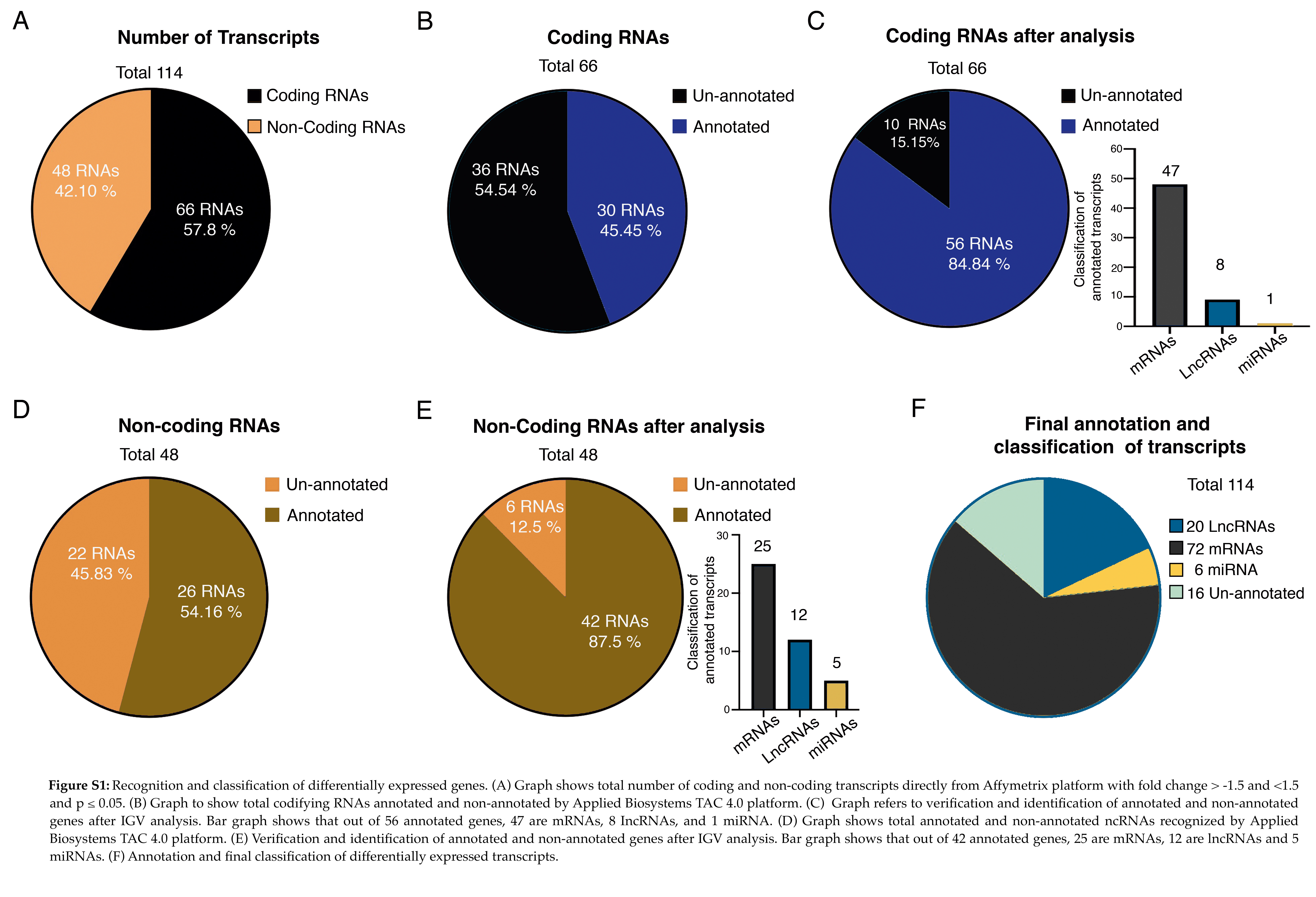

Supplement: Supplementary file 1 [file cancers-13-05602-s001.zip › Supplementary Figure 1 .jpg]

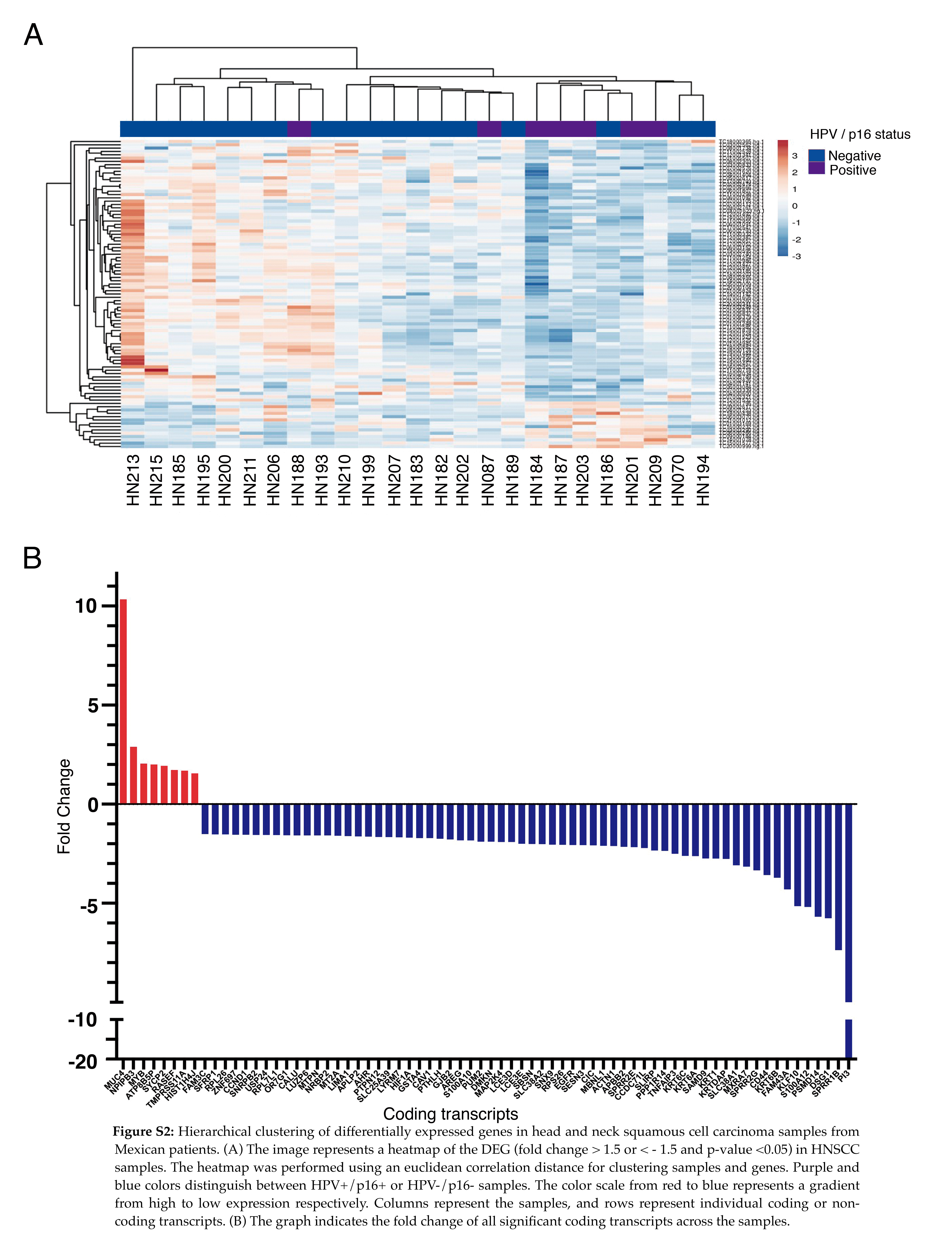

Supplement: Supplementary file 1 [file cancers-13-05602-s001.zip › Supplementary Figure 2.jpg]
